# Cost-benefit evaluation of advanced therapy lines in metastatic triple-negative breast cancer in Germany

**DOI:** 10.1186/s12962-024-00528-1

**Published:** 2024-03-08

**Authors:** Amelie Wickmann, Melina Sophie Kurte, Julia Jeck, Luisa Camacho, Dennis Klinkhammer, Florian Kron, Robert Dengler

**Affiliations:** 1 VITIS Healthcare Group, Cologne, Germany; 2 Faculty of Medicine, University of Duisburg-Essen, Essen, Germany; 3 Faculty of Medicine, Department I of Internal Medicine, University of Cologne, University Hospital Cologne, Cologne, Germany; 4 Faculty of Medicine, Center for Integrated Oncology (CIO ABCD), University of Cologne, University Hospital Cologne, Cologne, Germany; 5 FOM University of Applied Sciences, Essen, Germany; 6 Oncologic Healthcare Consulting GbR, Munich, Germany

**Keywords:** Triple-negative breast cancer, Efficiency frontier, Health-related quality of life, Sacituzumab-govitecan, Eribulin, Capecitabine, Vinorelbine

## Abstract

**Background:**

Triple-negative breast cancer (TNBC) is responsible for 10–20% cases of breast cancer and is resulting in rising healthcare costs. Thus, health-economic evaluations are needed to relate clinical outcomes and costs of treatment options and to provide recommendations of action from a health-economic perspective.

**Methods:**

We investigated the cost-benefit-ratio of approved treatment options in metastatic TNBC in Germany by applying the efficiency frontier approach. These included sacituzumab-govitecan (SG), eribulin, vinorelbine, and capecitabine. Clinical benefit was measured as (i) median overall survival (mOS) and (ii) health-related quality of life (HRQoL) in terms of time to symptom worsening (TSW). To assess medical benefits, literature was systematically reviewed in PubMed for (i) and (ii), respectively. Treatment costs were calculated considering annual direct outpatient treatment costs from a statutory healthcare payer perspective. It was intended that both, (i) and (ii), yield an efficiency frontier.

**Results:**

Annual direct outpatient treatment costs amounted to EUR 176,415.21 (SG), EUR 47,414.14 (eribulin), EUR 13,711.35 (vinorelbine), and EUR 3,718.84 (capecitabine). Systematic literature review of (i) and statistical analysis resulted in OS values of 14.3, 9.56, 9.44, and 7.46 months, respectively. Capecitabine, vinorelbine, and SG are part of the efficiency frontier including OS. The highest additional benefit per additional cost was determined for vinorelbine, followed by SG. Systematic review of (ii) revealed that no TSW data of TNBC patients receiving vinorelbine were available, preventing the presentation of an efficiency frontier including HRQoL.

**Conclusions:**

Vinorelbine is most cost-effective, followed by SG. Health-economic evaluations support decision-makers to assess treatment options within one indication area. In Germany, the efficiency frontier can provide decision support for the pricing of innovative interventions. Results of our analysis may thus guide reimbursement determination.

**Supplementary Information:**

The online version contains supplementary material available at 10.1186/s12962-024-00528-1.

## Background

Epidemiological studies report that triple-negative breast cancer (TNBC) is responsible for approximately 10–20% of all breast cancer cases [1–4], most frequently found in younger women [4]. TNBC is defined as estrogen-receptor (ER), progesterone-receptor (PR) and human epidermal growth factor receptor 2 (HER2) negative status [1–3]. Hence, TNBC patients are associated with unresponsiveness to both hormone and anti-HER2 therapy [1, 4]. In contrast to other breast cancer subtypes, metastatic TNBC is characterized by more aggressive course of disease and poorer survival [2], while treatment options are restricted [5]. In Germany, eribulin and the generic drugs vinorelbine and capecitabine are approved for treatment of metastatic TNBC in second-line setting. In 2021, sacituzumab govitecan (SG), a novel antibody-drug conjugate, was approved in this setting [6–8].

Treatment costs of malignant neoplasms in Germany amounted to 19.9 billion EUR in 2015, while further cost increase due to demographic developments and the introduction of innovative and high-priced therapies can be expected [9]. Hence, comparative health-economic evaluations are needed to relate clinical outcomes and costs of treatment options. The evaluation of survival and health-related quality of life (HRQoL) can provide additional information when comparing the efficiency of different treatments. However, health-economic evaluations of advanced therapy lines of metastatic TNBC in Germany are currently lacking. Thus, we aimed to investigate the cost-benefit-ratio of treatment options in metastatic TNBC in advanced therapy lines in Germany applying the efficiency frontier method from a healthcare payer perspective.

## Methods

### Theoretical framework: efficiency frontier

We compared costs and benefits of therapy lines for advanced metastatic TNBC using the efficiency frontier as methodological approach. This method is recommended by the German Institute for Quality and Cost Effectiveness in the Health Care Sector (IQWiG). The IQWiG is the German Health Technology Assessment authority and examines the benefits and harms of medical interventions, especially in the context of the early benefit assessment of new interventions, which is conducted by the Federal Joint Committee (*Gemeinsamer Bundesausschuss– G-BA)*. The efficiency frontier aims to graphically relate relative benefit and incurred costs of interventions within one indication. Visualized in a two-dimensional graph with costs on the x-axis and benefit on the y-axis, the interventions with respective higher efficiency are illustrated from left to right, forming the efficiency frontier. The additional benefit per additional cost is illustrated by the slope of the connecting line between two interventions [10]. We form the efficiency frontier under consideration of extended and absolute dominance. An intervention is absolutely dominated by another intervention if it poses higher costs with equal or reduced benefit. If an intervention provides a higher benefit along with higher costs, it cannot clearly be defined as inefficient. However, if a combination of two other interventions can achieve a higher benefit at lower costs, i.e., steeper slope of the efficiency frontier, the extendedly-dominated intervention is disregarded when plotting the efficiency frontier [10, 11].

### Clinical benefit

Our research question was defined according to the Population-Intervention-Comparison-Outcome (PICO) concept [10]. To date, approved therapy options for the indication of advanced metastatic TNBC in Germany include SG, eribulin, vinorelbine, and capecitabine. These are defined as appropriate comparative therapies by IQWiG in the latest early benefit assessment of SG [8], and thereby best depict clinical practice within this indication. Outcome was defined as clinical benefit, measured as (i) overall survival (OS) in terms of median OS and (ii) HRQoL in terms of time to symptom worsening (TSW) of global health status (GHS)/quality of life (QoL). HRQoL was assessed by using the GHS subscale of the European Organisation of Research and Treatment of Cancer Quality of Life Questionnaire (EORTC-QLQ) C30 and BR23 [12]. Although there is no uniformly accepted questionnaire for the assessment of HRQoL, the EORTC-QLQ-C30 is used in cancer patients worldwide, while the breast cancer EORTC-QLQ-BR23 is used as a disease specific extension [13, 14]. TSW was defined as the time until clinically meaningful deterioration by a specified threshold was observed for each patient-reported endpoint [15].

On May 2nd 2022, a systematic literature review in PubMed (MEDLINE) was conducted following IQWiG guidelines [10] and the PRISMA statement [16] for (i) and (ii), respectively. Pre-searches were absolved from April to May 2022. For (i), search terms such as *breast neoplasms*, *metastatic breast cancer*, *breast cancer* or *metastatic mamma carcinoma* were combined with the search terms *sacituzumab-govitecan*, *eribulin*, *vinorelbine*, and *capecitabine* and the search term *median survival* by using the Boolean Operator “AND”. For (ii), the search term was adapted by search terms such as *health related quality of life* and *patient reported outcome*, replacing *median survival*. For both, (i) and (ii), interventions were combined by the operator “OR”. For each term, synonyms, similar concepts, and different spellings were included by the operator “OR”. Medical subject headings (MeSH terms) were used if applicable. For (ii), further records were collected via other sources i.e., by examining relevant reference lists, due to rare evaluation of HRQoL in clinical studies [14]. For both, (i) and (ii), search hits were extracted with relevant information, i.e., author, title, journal, year, citation, create date, and DOI in Microsoft Excel, respectively, to systematically analyze identified articles.

For systematic literature reviews of (i) and (ii), in a first step, titles and abstracts identified from all searches and sources were assessed for eligibility criteria. Abstracts were excluded if treatment was administered in first-line setting, interventions or the patient population were not relevant to our research question, or OS or QoL were not evaluated. Secondly, if an abstract was considered to be potentially relevant, a full paper copy of the article was obtained. If available, it was reassessed for inclusion. Articles were considered if the study population included metastatic TNBC patients in advanced therapy setting with treatment of SG, eribulin, vinorelbine, or capecitabine, median OS or QoL measured by EORTC QLQ C30 or BR23 was reported for TNBC patients, and the article was available in English language. For systematic literature review of (i), literature reviews, pooled analyses, duplications of study populations, and case studies with less than 10 patients were excluded. For (ii), reviews were included for full paper assessment to identify further relevant primary studies. However, these reviews were then excluded from further analysis. Due to rare evaluation of HRQoL, bibliographies of all included articles were searched manually for additional references and other sources were screened to identify further literature. Here, also non-English literature was considered to extend possible findings. For both, (i) and (ii), studies that did not meet all criteria were considered inappropriate and were excluded; reasons for exclusion were documented. To avoid biases, two researchers individually conducted the literature reviews. Discrepancies were solved by discussion.

### Outpatient treatment costs

Based on the reference year 2022, annual direct outpatient treatment costs of advanced metastatic TNBC from healthcare payers’ perspective were calculated for each intervention, respectively. Costs are reported in Euro and differentiated for drug costs and medical services.

Drug costs for tumor medication including statutory discounts required by law (§ 130a German Social Code Book Five) were identified for SG, eribulin, capecitabine, and vinorelbine in the decision on early benefit assessment by the Federal Joint Committee [8]. If price ranges were reported, mean values were calculated. Costs for drug preparation were identified according to Annex 3 to the Contract on Pricing of Substances and Preparations of Substances [17]. Concomitant medication (i.e., antiemetics, antiallergics, corticosteroids, etc.) were identified according to applicable laws and a drug database (*Lauer-Taxe*) [18, 19].

In Germany, medical services provided in outpatient care and reimbursed by statutory health insurances are defined by the valuation committee and listed in the uniform valuation scale (*Einheitlicher Bewertungsmaßstab– EBM*) as fee schedule items (*Gebührenordnungsposition– GOP*) [20]. Medical services for oncologic patients are additionally reimbursed quarterly via oncology agreements on federal state level also using fee schedule items [21]. The costs for medical services were calculated as amount of reimbursement by statutory health insurance. Based on the uniform valuation scale from 2022, relevant fee schedule items for treatment of metastatic TNBC patients and administration of drugs were identified for each intervention, respectively, to calculate costs for medical services [20]. Further, relevant mean fee schedule items of the federal oncology agreement were taken into account [21].

### Statistical analysis

As clinical benefit was considered in terms of (i) median OS and (ii) TSW of GHS/QoL, it was intended that both medical benefits each yield an efficiency frontier. For interventions with more than one result for (i) or (ii), we applied bootstrap method in R studio (posit cloud) [22] to obtain the most probable point estimates of the varying median OS estimators using 1,000 repetitions for simulation [23, 24]. For graphical illustration of the efficiency frontier, the mean of these results was used as point estimates for (i) or (ii), respectively. Additionally, to account for uncertainty, the standard deviation was estimated for interventions with more than one result and graphically illustrated by vertical bars.

### Sensitivity analysis

To verify our results for point estimators regarding clinical benefit, a Monte-Carlo-Simulation was conducted in R studio (posit cloud) [22] for all interventions with more than one result for (i) or (ii), respectively. Based on the assumption of Stollenwerk et al. [25] we assumed a normal distribution. Monte-Carlo-Simulation was performed using 1,000 repetitions. The results were illustrated graphically as histograms. Costs were considered invariable and were therefore not probabilistic.

## Results

### Clinical benefit

**Fig. 1:**
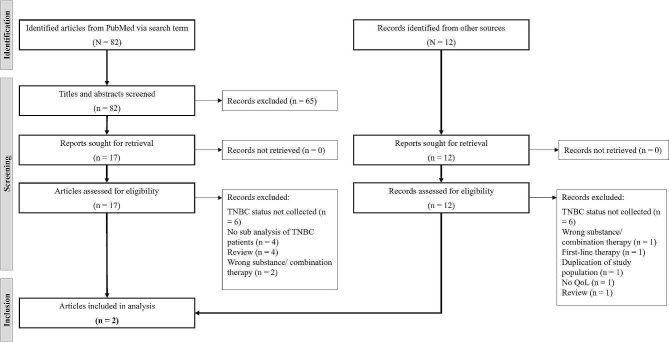
PRISMA flow diagram for outcome (i) median OS

For overall survival, in total, 833 records were identified in PubMed, shown in Fig. 1. By screening titles and abstracts 695 records were excluded according to pre-defined criteria and 138 records were sought for retrieval. As 18 records were not publicly accessible, 120 articles were assessed for eligibility. Most records were excluded due to absent sub-analysis of TNBC patients (*n* = 43) or lacking TNBC status (*n* = 34). Summarized under “other”, nine records were excluded because they were pooled analyses, reviews or case studies, they considered combination therapies, we identified duplications of study populations, or median OS was not reported. The systematic review resulted in 15 articles to be included into our analysis of clinical benefit in terms of survival. Study characteristics for SG [6, 26], eribulin [27–38], capecitabine [28, 34], and vinorelbine [39] are summarized in Table 1.

**Table 1:** Results of the systematic literature review regarding median OS

Authors	Title	Year	Journal	Intervention	Population [n]	Median OS
Bardia, A. et al. [6]	*Sacituzumab Govitecan in Metastatic Triple-Negative Breast Cancer*	*2021*	*N Engl J Med*	*SG*	*267*	*11.8 months*
Bardia, A. et al. [26]	*Efficacy and Safety of Anti-Trop-2 Antibody Drug Conjugate Sacituzumab Govitecan (IMMU-132) in Heavily Pretreated Patients With Metastatic Triple-Negative Breast Cancer*	*2017*	*J Clin Oncol*	*SG*	*69*	*16.6 months*
Sari, M. et al. [36]	*Eribulin monotherapy in heavily pretreated metastatic breast cancer patients in real life*	*2020*	*Indian J Cancer*	*Eribulin*	*7*	*10 months*
Krasniqi, E. et al. [27]	*Second-line Eribulin in Triple Negative Metastatic Breast Cancer patients. Multicentre Retrospective Study: The TETRIS Trial*	*2021*	*Int J Med Sci*	*Eribulin*	*44*	*11.9 months*
Kazmi, S. et al. [28]	*Overall survival analysis in patients with metastatic breast cancer and liver or lung metastases treated with eribulin, gemcitabine, or capecitabine*	*2020*	*Breast Cancer Res Treat*	*Eribulin Capecitabine*	*66 (Eribulin)* *20 (Capecitabine)*	*7.0 months (Eribulin)* *5.5 months (Capecitabine)*
Ates, O. et al. [37]	*Efficacy and safety of eribulin monotherapy in patients with heavily pretreated metastatic breast cancer*	*2016*	*J BUON*	*Eribulin*	*7*	*3 months*
Mougalian, SS. et al. [29]	*Clinical benefit of treatment with eribulin mesylate for metastatic triple-negative breast cancer: Long-term outcomes of patients treated in the US community oncology setting*	*2018*	*Cancer Med*	*Eribulin*	*127*	*14.7 months*
Miyoshi, Y. et al. [30]	*High absolute lymphocyte counts are associated with longer overall survival in patients with metastatic breast cancer treated with eribulin-but not with treatment of physician’s choice-in the EMBRACE study*	*2020*	*Breast Cancer*	*Eribulin*	*27* 65*	*9.5 months* 9.5 months**
Valerio, M.R. et al. [31]	*Eribulin Mesylate for the Treatment of Metastatic Hormone-refractory and Triple-negative Breast Cancer: A Multi-institutional Real-world Report on Efficacy and Safety*	*2021*	*Am J Clin Oncol*	*Eribulin*	*38*	*10.8 months*
Decker, T. et al. [39]	*VicTORia: a randomised phase II study to compare vinorelbine in combination with the mTOR inhibitor everolimus versus vinorelbine monotherapy for second-line chemotherapy in advanced HER2-negative breast cancer*	*2019*	*Breast Cancer Res Treat*	*Vinorelbine*	*12*	*9.44 months*
Pedersini, R. et al. [38]	*Eribulin in Heavily Pretreated Metastatic Breast Cancer Patients in the Real World: A Retrospective Study*	*2018*	*Oncology*	*Eribulin*	*8*	*7.43 months*
Aogi, K. et al. [32]	*A phase II study of eribulin in Japanese patients with heavily pretreated metastatic breast cancer*	*2012*	*Ann Oncol*	*Eribulin*	*22*	*10.68 months*
Mougalian, SS. et al. [33]	*Effectiveness of Eribulin in Metastatic Breast Cancer: 10 Years of Real-World Clinical Experience in the United States*	*2021*	*Adv Ther*	*Eribulin*	*256*	*9.8 months*
Twelves, C. et al. [34]	*Subgroup Analyses from a Phase 3, Open-Label, Randomized Study of Eribulin Mesylate Versus Capecitabine in Pretreated Patients with Advanced or Metastatic Breast Cancer*	*2016*	*Breast Cancer (Auckl)*	*Eribulin* *Capecitabine*	*106 (Eribulin)* *102 (Capecitabine)*	*15.2 months (Eribulin)* *9.2 months (Capecitabine)*
Chan, A. et al. [35]	*Incorporation of eribulin in the systemic treatment of metastatic breast cancer patients in Australia*	*2022*	*Asia Pac J Clin Oncol.*	*Eribulin*	51***	*4.3 months*

* Two arms received eribulin

** We included the average into the efficiency frontier calculation

*** Number of total TNBC population, the exact number of patients evaluated for late-use not provided

**Fig. 2:**
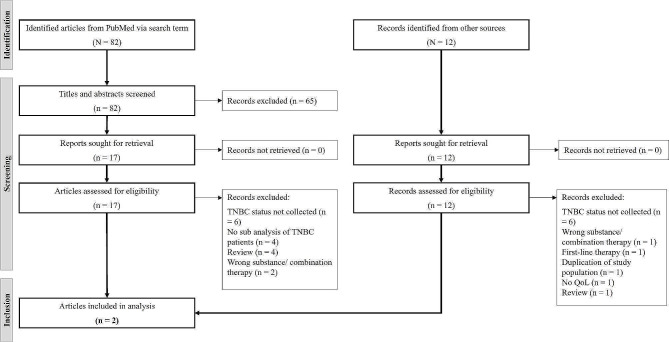
PRISMA flow diagram for outcome (ii) HRQoL

For HRQoL, 82 records were identified in total, from which 65 records were excluded, shown in Fig. 2. Sixteen records were not eligible to our research question. Of these, six records were excluded due to missing TNBC status, four did not sub-analyze the TNBC population, and four were reviews. Further twelve records were identified from other sources, of which eleven were excluded. The systematic review for HRQoL therefore resulted in two sources reporting HRQoL data in terms of TSW on SG, eribulin, and capecitabine [40, 41] using the EORTC QLQ C30 or BR23 questionnaire. Data on vinorelbine could not be identified. The results on median TSW of GHS/QoL and of important function and symptom items can be obtained from Table 2. Median TSW of GHS/QoL amounted to 2.8, 6.2, and 6.0 months for SG, eribulin, and capecitabine, respectively. Baseline scores were comparable along all interventions.

**Table 2:** Results of the systematic literature review regarding median TSW in months

	Sacituzumab Govitecan [41]	Eribulin [40]	Capecitabine [40]	Vinorelbine
GHS/QoL	*2.80*	*6.2*	*6.0*	*--*
Physical functioning	*5.90*	*--*	*--*	*--*
Role functioning	*2.10*	*--*	*--*	*--*
Emotional functioning	*5.90*	*--*	*--*	*--*
Cognitive functioning	*3.30*	*--*	*--*	*--*
Social functioning	*3.30*	*--*	*--*	*--*
Body image	*--*	*6.7*	*6.0*	*--*
Future perspective	*--*	*6.0*	*4.8*	*--*
Systemic therapy side-effects	*--*	*4.9*	*7.2*	*--*
Fatigue	*1.60*	*8.9*	*6.1*	*--*
Nausea/vomiting	*2.10*	*9.9*	*6.5*	*--*
Dyspnea	*6.90*	*--*	*--*	*--*
Pain	*4.90*	*8.1*	*5.4*	*--*
Diarrhea	*2.00*	*11.6*	*6.6*	*--*
Appetite loss	*3.00*	*--*	*--*	*--*
Constipation	*3.60*	*--*	*--*	*--*
Insomnia	*4.10*	*--*	*--*	*--*

GHS - Global health status; QoL - Quality of life

### Outpatient treatment costs

Shown in Table 3, we identified fee schedule items and corresponding Euro values as well as their frequency for each intervention, respectively. Drug costs were highest for SG with EUR 173,245.08 and lowest for capecitabine with EUR 2,601.84. We identified costs for reimbursement of medical services comprising fee schedule items such as basic flat rates, medical therapy, infusion, outpatient supervision, imaging (computed tomography), and laboratory services. Relevant fee schedule items defined by oncology agreement were identified, including oral or intravascular therapy. Costs for medical services were highest for SG with EUR 3,170.13 and lowest for capecitabine with EUR 1,117.00. In total, we identified annual direct outpatient treatment costs of EUR 176,415.21, EUR 47,414.14, EUR 3,718.84, and EUR 13,711.35 for SG, eribulin, capecitabine, and vinorelbine, respectively.

**Table 3:** Annual direct outpatient treatment costs of metastatic TNBC

			Sacituzumab govitecan		Eribulin		Capecitabine		Vinorelbine	
Drug Costs [8, 17–19]		Value in EUR	Quantity	Costs in EUR	Quantity	Costs in EUR	Quantity	Costs in EUR	Quantity	Costs in EUR
Tumor medication				*167,239.06*		*39,892.63*		*2,454.96*		*7,789.39*
Preparation		*81.00*	*32*	*2,592.00*	*32*	*2,592.00*	*0*	*-*	*48*	*3,888.00*
Concomitant medication				*3,414.02*		*3,163.23*		*146.88*		*146.88*
TOTAL				*173,245.08*		*45,647.86*		*2,601.84*		*11,824.27*
Costs for medical services [20, 21]	*GOP*	*Value in EUR*	*Quantity*	*Costs in EUR*	*Quantity*	*Costs in EUR*	*Quantity*	*Costs in EUR*	*Quantity*	*Costs in EUR*
Basic flat rate	*13,491*	*35.38*	*4*	*141.52*	*4*	*141.52*	*4*	*141.52*	*4*	*141.52*
Additional flat rate	*13,500*	*21.52*	*4*	*86.08*	*4*	*86.08*	*4*	*86.08*	*4*	*86.08*
Medical therapy	*13,502*	*19.94*	*4*	*79.76*	*4*	*79.76*	*4*	*79.76*	*4*	*79.76*
Infusion	*02100*	*7.55*	*0*	*-*	*32*	*241.60*	*0*	*-*	*48*	*362.40*
Outpatient supervision (2 h)	*01510*	*49.91*	*31*	*1,547.21*	*0*	*-*	*0*	*-*	*0*	*-*
Outpatient supervision (4 h)	*01511*	*98.24*	*1*	*98.24*	*0*	*-*	*0*	*-*	*0*	*-*
Computed tomography	*34,340*	*65.46*	*4*	*261.84*	*4*	*261.84*	*4*	*261.84*	*4*	*261.84*
Laboratory										
*Blood count*	*32,120*	*0.50*	*52*	*26.00*	*52*	*26.00*	*52*	*26.00*	*52*	*26.00*
Blood serum	*32,056–32,079*	*2.50*	*12*	*30.00*	*12*	*30.00*	*12*	*30.00*	*12*	*30.00*
Active tumor disease care	*86,512*	*26.16*	*4*	*104.64*	*4*	*104.64*	*4*	*104.64*	*4*	*104.64*
Oral therapy	*86,520*	*96.79*	*0*	*-*	*0*	*-*	*4*	*387.16*	*0*	*-*
Intravascular therapy	*86,516*	*198.71*	*4*	*794.84*	*4*	*794.84*	*0*	*-*	*4*	*794.84*
TOTAL				*3,170.13*		*1,766.28*		*1,117.00*		*1,887.08*
TOTAL COSTS				*176,415.21*		*47,414.14*		*3,718.84*		*13,711.35*

GOP - Fee schedule item (Gebührenordnungsposition)

### Statistical analysis

After applying the bootstrap method for interventions with more than one result, OS point estimates and respective standard deviation for SG, eribulin, and capecitabine were 14.3 (2.4), 9.56 (3.61), and 7.46 (1.85) months, respectively. As only one paper reporting OS was maintained for vinorelbine, the median OS of 9.44 [39] was used as point estimate and no standard deviation could be calculated. For HRQoL, no statistical analysis was feasible.

### Efficiency frontier

**Fig. 3:**
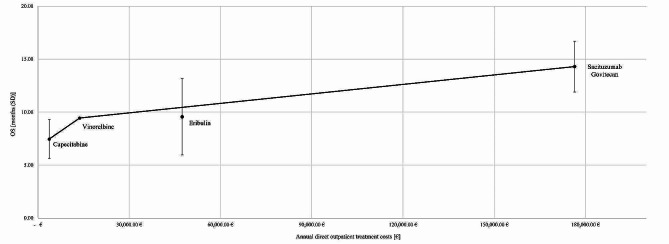
Efficiency frontier of advanced therapy lines in advanced metastatic TNBC

To relate clinical benefit and treatment costs of advanced therapy lines in advanced metastatic TNBC, the results regarding clinical benefit are combined with the results of the cost calculation. Our literature review on HRQoL did not provide sufficient clinical value data on vinorelbine. Thus, the underlying data did not meet the methodological requirements to plot an efficiency frontier. Figure 3 shows the efficiency frontier using point estimates for OS for advanced therapy lines in advanced metastatic TNBC. With increasing OS, capecitabine, vinorelbine, and SG form the efficiency frontier and are thereby efficient treatment alternatives. Eribulin is not part of the efficiency frontier due to the extended dominance, i.e., it is not clearly inefficient. The vertical bars around the point estimates of the respective intervention show the uncertainty of the point estimates. The standard deviation is highest for eribulin. The slope of the connecting line from capecitabine to vinorelbine is steepest, followed by the connecting line from vinorelbine to SG. Hence, the additional benefit per additional cost is highest for vinorelbine, followed by SG. Based on the underlying efficiency frontier, the newest intervention SG is of greater medical benefit (14.3 months OS) compared to the other interventions, while generating the highest costs.

### Sensitivity analysis

Mean point estimates for clinical benefit in terms of OS and respective standard deviations amounted to 14.26 (3.43), 9.59 (3.72), and 7.4 (2.65) for SG, eribulin, and capecitabine, respectively. These results are comparable to our findings using bootstrap method. For vinorelbine, as only one study was available, Monte-Carlo-Simulation was unfeasible. The histograms for Monte-Carlo-Simulation of SG, eribulin, and capecitabine for clinical benefit in terms of OS are shown in Additional file 1 to 3, respectively.

## Discussion

In this analysis, we examined advanced therapy lines in metastatic TNBC relating clinical benefit and treatment costs using the efficiency frontier as methodological approach from a health-economic perspective. We aimed to evaluate the cost-benefit ratios of the underlying interventions in metastatic TNBC, considering OS and HRQoL as clinical benefit.

Using OS as outcome parameter, we showed that capecitabine, vinorelbine, and SG form the efficiency frontier. Vinorelbine is the most cost-effective treatment alternative due to highest additional benefit per additional cost, followed by SG. Our results should be viewed in consideration of the generic characteristics of vinorelbine and capecitabine, as they are thereby less costly than innovative, patented drugs, such as SG and eribulin. Due to the extended dominance, eribulin is not part of the efficiency frontier, i.e., it is not clearly inefficient. Despite the higher costs, SG has longest OS, followed by eribulin, vinorelbine, and capecitabine, showing the shift in innovation within the indication of metastatic TNBC. The lack of studies evaluating HRQoL prevented the determination of an efficiency frontier using HRQoL as outcome parameter. Based on our information, the ASCENT study is the only trial to compare our underlying interventions for TNBC in a randomized controlled trial (RCT) regarding OS [6]. Results on median OS of this study population in respect to the interventions eribulin, vinorelbine and capecitabine have been published by O’Shaughnessy et al. [42], showing significantly shorter OS rates for all interventions compared to our aggregated findings. However, the differences on OS along all interventions are somewhat comparable. We did not include these findings into our analysis of OS as they have not been published in a peer-reviewed journal up to the date of our literature review.

As described by Stollenwerk et al. [25], the efficiency approach can support policy makers by determining the trade-off between costs and benefits within one indication and provide guidance for estimating reimbursement prices for new interventions in Germany [25]. Therefore, the last segment of the efficiency frontier is extrapolated to indicate beneficial or unbeneficial cost-benefit ratios by their position below or above the extrapolated segment of the efficiency frontier. If SG was considered a new intervention, capecitabine, vinorelbine, and eribulin would form the efficiency frontier. The segment between vinorelbine and eribulin would be linearly extrapolated and SG could be reimbursed at the indicated price due to a better cost-benefit-ratio [10].

In the United Kingdom (UK), the National Institute for Health and Care Excellence (NICE) assesses costs and benefits of interventions [43]. While the efficiency frontier does not allow cross-therapeutic area comparisons, the quality adjusted life year (QALY) approach, especially used by the NICE, refers to one threshold across all therapeutic areas [43]. From US perspective, an economic evaluation of SG by Chen et al. [44] showed an incremental cost effectiveness ratio of USD 494,479 per QALY, an increase of 0.35 QALYs and extra costs of USD 175,393 when comparing SG to chemotherapy [44]. In Germany, the use of QALYs is criticized by IQWiG due to ethical and methodological reasons [43], especially considering the determination of thresholds [10]. In the context of evaluating disease- and treatment-related changes, IQWiG considers patient-relevant outcomes such as mortality, morbidity, and HRQoL [10]. By means of our twofold analysis of clinical benefit, we aimed to include not only mortality in terms of OS but also HRQoL. However, HRQoL of TNBC patients is scarcely reported in literature. Most studies did not investigate HRQoL data at all or the tools used for assessment varied significantly. For SG, HRQoL data was published as congress contribution by Loibl et al. [45], however, not as complete as in the dossier for early benefit assessment [41]. As TNBC exhibits significant differences within breast cancer in respect to age, stage, prognosis, and therapy regimens, all of which influence QoL patterns, more high quality prospective clinical trials are warranted to meet the need of this subgroup of patients. The use of HRQoL scales is also relevant in other health-economic evaluations. Tremblay et al. [46] conducted an economic evaluation of eribulin for metastatic breast cancer including HRQoL data to illustrate utility of progression-free survival and progression [46].

Our analysis is not without limitations. To collect and critically appraise available knowledge for the defined research question, systematic literature reviews were conducted. However, cross-study comparisons of treatment arms were needed to compare findings on clinical benefit within the indication of metastatic TNBC using the methodological approach of the efficiency frontier. Differences in baseline characteristics of the underlying study population, the use of previous therapies, and differences in population size have not been considered, which may limit the results (see Additional file 4 for a comparison of baseline characteristics of the study populations). Future research should include statistical analysis to account for cross-study comparisons of treatment arms, using for example matching-adjusted indirect comparison (MAIC) or propensity scores [47, 48]. However, this was not feasible, as no individual patient data were available when conducting our analysis. The mentioned limitations notwithstanding, cross-study comparisons with aggregated patient data are nonetheless used, also in breast cancer research [49]. In our analysis, the uncertainty of the point estimate for eribulin limits the interpretation of our efficiency frontier. Uncertainty in the considered parameters can affect the location of the efficiency frontier. Hence, it is not a rigid construct, especially considering potential variation when taking other studies into account. In the context of current clinical developments, future research could address the limitations of the efficiency frontier approach regarding the comparability of different interventions and the restricted clinical evidence in small subgroups of patients.

For both outcomes, literature evaluating outcomes of TNBC study populations were rare. Especially studies evaluating OS of TNBC patients treated with capecitabine or vinorelbine were lean. Due to the novelty of SG, the number of studies evaluating SG to be included in our analysis was restricted. Theses aspects could limit the transferability of our results, especially, as statistical analyses were not feasible for interventions with only one outcome and the efficiency frontier is based on some studies with small population sizes. Overall, HRQoL is only scarcely evaluated in TNBC patients. Further studies should assess HRQoL in TNBC patients, especially considering the relevance of health-economic evaluations taking important HRQoL measures into account.

In our analysis, inpatient treatment costs were disregarded. Future research could include inpatient treatment costs for TNBC patients, such as adverse event management costs.

## Conclusions

Our analysis assessed cost-benefit ratios of advanced therapy lines for metastatic TNBC. We related clinical benefit in terms of OS with annual direct outpatient treatment costs applying the efficiency frontier as methodological approach. The results showed the need for additional clinical studies evaluating HRQoL to allow further cost-benefit evaluations of metastatic TNBC interventions using HRQoL as outcome measure. Although the efficiency frontier method is recommended by IQWiG, it is rarely implemented in German and international health-economic practice up to now. Nonetheless, it can serve as decision support for pricing of innovative interventions for statutory health insurances. Results of our analysis may thus assist decision makers in reimbursement determination. However, other aspects should be considered in future research, such as the budgetary impact on the entire healthcare system and the costs along the patient journey, especially in the case of advanced therapies.

### Electronic supplementary material

Below is the link to the electronic supplementary material.

Supplementary Material 1

## Abbreviations

TNBC: Triple-negative breast cancer
ER: Estrogen-receptor
PR: Progesterone-receptor
HER2: Human epidermal growth factor receptor 2
SG: Sacituzumab govitecan
HRQoL: Health-related quality of life
IQWiG: German Institute for Quality and Cost Effectiveness in the Health Care Sector
PICO: Population-Intervention-Comparison-Outcome
OS: Overall survival
TSW: Time to symptom worsening
GHS: Global health status
QoL: Quality of life
EORTC-QLQ: European Organisation of Research and Treatment of Cancer Quality of Life Questionnaire
RCT: Randomized controlled trial
UK: United Kingdom
NICE: National Institute for Health and Care Excellence
QALY: Quality adjusted life year
MAIC: Matching-adjusted indirect comparison
