# Supplementary material for: Cost-benefit evaluation of advanced therapy lines in metastatic triple-negative breast cancer in Germany
Source: Cost Eff Resour Alloc. 2024 Mar 8;22:21. doi: 10.1186/s12962-024-00528-1 (PMC10924420; doi:10.1186/s12962-024-00528-1)
Supplement: Supplementary file 1 — Supplementary Material 1 [file 12962_2024_528_MOESM1_ESM.docx]

**ADDITIONAL FILES**


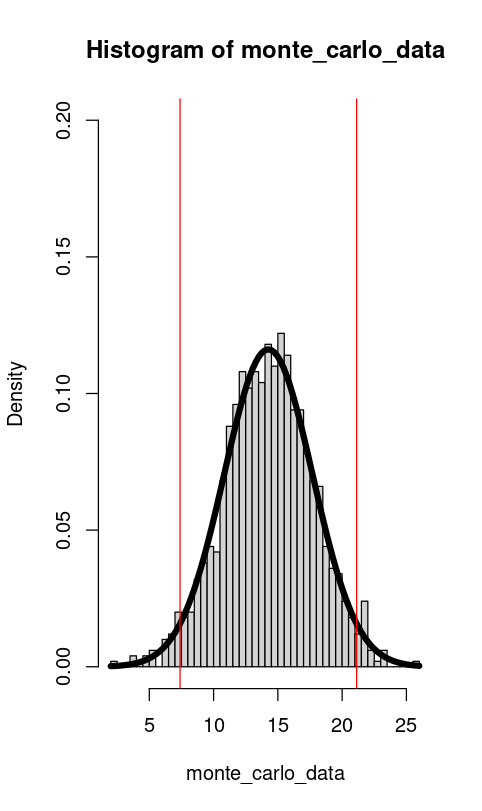
Additional file 1 Histogram of Monte-Carlo-Simulation for SG


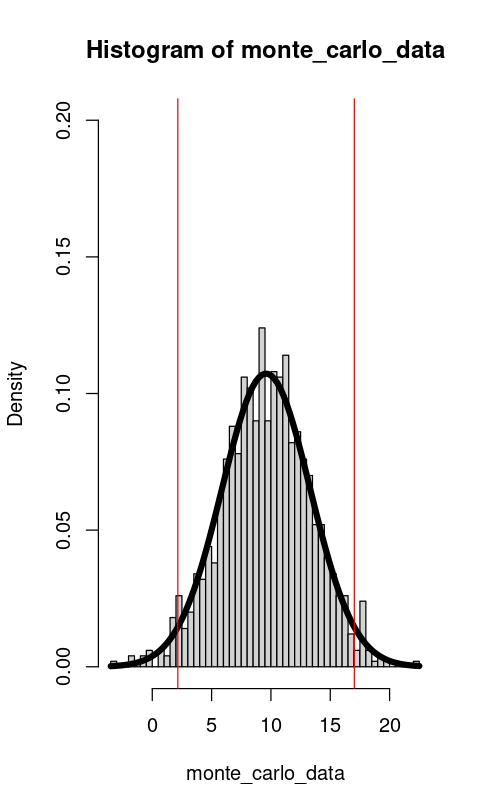
 Additional file 2 Histogram of Monte-Carlo-Simulation for eribulin


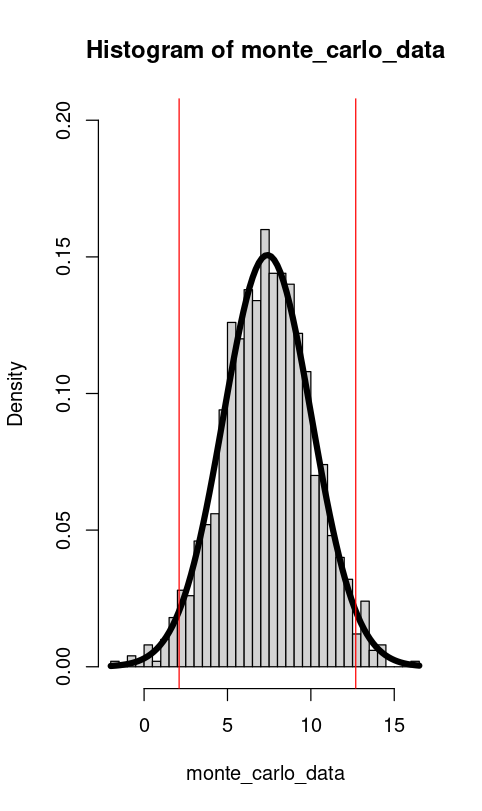
 Additional file 3 Histogram of Monte-Carlo-Simulation for capecitabine

Additional file 4 Patient characteristics

| *Authors*  *[Reference]* | *Total study population [n]* | *Study population Triple negative [n]* | *Age [median (range)]* | *Gender* | *Lines of prior therapies [median (range)]* | *ECOG performance-status* | *Number of metastatic sites* | *Germline BRCA1 or BRCA2 mutation status* |
| --- | --- | --- | --- | --- | --- | --- | --- | --- |
| *Bardia, A. et al. [6]* | *529* | *529* | *54 (27-82)* | *233 female, 2 male*  *(Without Brain metastasis)* | *Not specified* | *0 = 108*  *1 = 127*  *(Without Brain metastasis)* | *Not specified* | *BRCA 1/2 mutation*  *negative = 133*  *positive = 16*  *(Without Brain metastasis)* |
| *Bardia, A. et al. [26]* | *69* | *69* | *56 (31-81)* | *68 female, 1 male* | *5 (1-12)* | *0 = 23*  *1 = 46* | *Lymph node = 43*  *Lung = 35*  *Liver = 30*  *Chest = 28*  *Bone = 21*  *Skin = 7* | *All BRCA1* |
| *Sari, M. et al. [36]* | *28* | *7* | *52.5 (29-72)* | *All female* | *4 (1–7)* | *1 (0-2) [median, range]* | *3 (1-4)* | *Not specified* |
| *Krasniqi, E. et al. [27]* | *44* | *40* | *51 (35-81)* | *All female* | *Not specified* | *0 = 17*  *1 = 24*  *2= 2*  *unknown = 1* | *Number of metastatic sites*  *1 = 13*  *2 = 20*  *> 2= 11* | *BRCA 1/2 mutation*  *Yes = 1*  *No = 26 Unknown = 17* |

| *Kazmi, S. et al. [28]* | *443* | *66 (Eribulin) 20 (Capecitabine)*  *36 (Gemcitabin)* | *55.0 (8.63) (Eribulin)*  *55.9 (9.49) (Gemcitabin) 55.5 (9.7) (Capecitabine* | *All female* | *Not specified* | *0= 39*  *1=119*  *2=53*  *3= 12*  *4= 1*  *unknown = 5*  *(Eribulin)*  *0= 15*  *1= 63*  *2= 43*  *3= 9*  *4=0*  *Unknown= 4*  *(Gemcitabine)*  *0= 23*  *1= 34*  *2= 9*  *3= 5*  *4= 1*  *Unknown= 8*  *(Capecitabine)* | *Not specified* | *Not specified* |
| --- | --- | --- | --- | --- | --- | --- | --- | --- |
| *Ates, O. et al. [37]* | *66* | *7* | *50 (28-67)* | *All female* | *4 (2-7)* | *0 = 29*  *1 = 30*  *2 = 7* | *Bone = 58*  *Lung = 51*  *Brain = 19*  *Liver = 38*  *Local recurrence = 7* | *Not specified* |
| *Mougalian, SS. et al. [29]* | *252* | *127* | *53* | *All female* | *4 (1-6)* | *0 = 40*  *1 = 156*  *2 = 56*  *3/4 = 0* | *Bone=151*  *Liver=110*  *Lymph nodes=114*  *Lung=127*  *Brain=11*  *Other=14* | *Not specified* |

| *Miyoshi, Y. et al [30]* | *751* | *92* | *<65 years = 604*  *≥65 years = 147* | *Not specified* | *≤ 3 = 351*  *> 3 = 397* | *0 = 317*  *≥ 1 = 423* | *Number of organs involved*  *≤2 =531*  *>2 = 212* | *Not specified* |
| --- | --- | --- | --- | --- | --- | --- | --- | --- |
| *Valerio, M.R. et al. [31]* | *90* | *38* | *62 (38-72)* | *All female* | *Not specified* | *0-1 = 82*  *2 = 8* | *Sites of disease Breast = 12 Node/soft tissue = 34*  *Lung/pleura = 27*  *Liver = 36*  *Bone = 45*  *Brain = 5* | *Not specified* |
| *Decker, T. et al. [39]* | *133* | *60* | *61.9 (Arm1, n = 68))*  *60.7 (Arm2, n = 65)* | *All female* | *Not specified* | *0 = 66*  *1 = 60*  *2 = 6* | *1 = 35*  *2 = 50*  *≥3 = 45* | *Not specified* |
| *Pedersini, R. et al. [38]* | *53* | *8* | *62 (30–79)* | *All female* | *4 (2–7)* | *0-1 = 41*  *2 = 12* | *Sites of metastases*  *Liver = 30*  *Lung = 11*  *Bone = 9*  *Others = 3* | *Not specified* |
| *Aogi, K. et al. [32]* | *80* | *22* | *54.0 (31–72)* | *All female* | *3 (1-5)* | *0 = 58*  *1 = 21*  *2 = 1* | *No. of organs involved,*  *1 = 7*  *2 = 15*  *3 = 23*  *4 = 21*  *5 = 14* | *Not specified* |

| *Mougalian, SS. et al. [33]* | *513* | *256* | *57.0 (16.0)* | *All female* | *3=225*  *4=25*  *5=6* | *0/1 = 168*  *≥ 2 =88*  *Unknown = 0* | *Sites of metastases at initiation of eribulin (n, %) Adrenal gland = 26*  *Brain = 15*  *Local lymph node(s)= 40*  *Gastrointestinal system = 1*  *Genitourinary system = 4*  *Ovary = 5*  *Liver = 159*  *Lung = 179*  *Lytic or mixed lytic-blastic bone = 33*  *Pleura, pericardial, and/or peritoneal cavity = 16*  *Othera = 3* | *Not specified* |
| --- | --- | --- | --- | --- | --- | --- | --- | --- |
| *Twelves, C. et al. [34]* | *438 (Eribulin)*  *444 (Capecitabine)* | *106 (Eribulin) 102 (Capecitabine)* | *Not specified* | *All female* | *Not specified* | *Not specified* | *Not specified* | *Not specified* |
| *Chan, A. et al. [35]* | *266* | *51* | *49.8 (25–77)* | *Not specified* | *4 (1–14)* | *Not specified* | *1 = 54 (20)*  *2 = 85 (32)*  *3 = 55 (21)*  *≥ 4 = 72 (27)* | *Not specified* |

Abbreviations: ECOG, Eastern Cooperative Oncology Group; PS, performance status
